# Prevalence, Etiology, and Types of Dental Trauma in Self-Assessment of 18-Year-Olds in Poland

**DOI:** 10.3390/ijerph191912924

**Published:** 2022-10-09

**Authors:** Dorota Olczak-Kowalczyk, Anna Turska-Szybka, Marcin Studnicki, Jacek Tomczyk

**Affiliations:** 1 Department of Pediatric Dentistry, Medical University of Warsaw, 02-091 Warsaw, Poland; 2 Department of Biometry, Institute of Agriculture, Warsaw University of Life Sciences, 02-787 Warsaw, Poland; 3 Institute of Biological Sciences, Cardinal Stefan Wyszynski University, 01-815 Warsaw, Poland

**Keywords:** traumatic dental injuries, youth, questionnaire, Poland

## Abstract

The aim of the study is to determine the occurrence and etiology of traumatic dental injuries of permanent teeth and to evaluate the relationship between diagnosed injuries and selected socio-economic indicators. In total, 1741 students aged 18 years, representing all the regions of Poland, took part in the survey. Questionnaires for monitoring studies were prepared in accordance with the criteria of the World Health Organization. Among the respondents, tooth trauma was reported by 18% of adolescents. The most common trauma occurred during other activities (e.g., playing) (64%). The most common direct cause of injury was a fall (43%) or a collision with an object or another person (36%). Risk of injury was increased by a low level of parents’ education and poor financial situation of the family. Playing sports is important in the etiology of injuries. The incidence of injuries was highest in school, demonstrating the need for school education in injury prevention and first aid. Occurrence of injuries, their causes, therapy, and post-traumatic complications were similar in respondents of both genders, which can be explained by secularization trends. Risk of injury was increased by parents’ low level of education and poor economic status. The research demonstrates the need for universal education on treatment of dental injuries.

## 1. Introduction

Traumatic dental injuries (TDI) and their health, economic, and psychosocial consequences are a serious problem for children during development. Treatment of TDI and the resulting complications is time-consuming, costly, long-term, and, in many cases, burdened with a high risk of failure. Therefore, treatment of traumatic injuries places a heavy burden on the health care system, patients, and their families [1,2].

It is estimated that about 20% of adults have suffered a permanent tooth injury and 17% of people under the age of 18 [3,4,5,6]. The incidence of dental trauma in different regions of the world varies, ranging from 2 to 59% [7]. Such a high degree of diversity results, inter alia, from different research methodologies and the influence of various socio-economic and cultural factors in the studied populations [3,6].

Few of the studies assessing the prevalence and etiology of tooth injuries are prospective and longitudinal. Usually, they are cross-sectional clinical studies of a selected age group. Many of these studies are retrospective in nature and present the results of an analysis of patients’ medical records. Both prospective and retrospective studies carry the risk of underestimating data. These studies are based on a group of people who came to the dentist for treatment, excluding those who had a tooth injury and did not receive professional care [3,4].

Cross-sectional research is based on a clinical evaluation of the effects of the injuries visible on the day of the study. Thus, it does not judge previous episodes, such as subluxation, luxation, or root fractures. Research based on self-report also carries a risk of underestimation due to forgetting an episode of trauma or its type [8]. There are also reports about the substantial difference between clinical and self-reported findings. It should be emphasized that the questions asked by researchers about dental injuries in the questionnaires are usually not consistent with the possibilities of assessment in a clinical trial [9].

The results of studies analyzing the etiology of injuries to permanent teeth also vary [10]. Some researchers have reported a relationship between permanent tooth injuries and socio-economic factors. These economic and social factors influence quality of life and manner of spending free time. However, the results of studies are divergent, which means that the significance of these factors is still unclear [11,12,13,14]. Meanwhile, knowledge about the factors contributing to tooth trauma and influencing the therapeutic approach is crucial in planning activities aimed at reducing the risk of tooth trauma and its consequences. Therefore, there is a need for further research to isolate the factors related to TDI. 

The aim of this study was to evaluate self-reported dental trauma of permanent teeth and types of trauma among adolescents aged 18 years. The study also assessed the etiology of dental trauma, receipt of dental treatment, and the relationship between self-reported injuries and selected socio-economic indicators. We assumed that factors such as gender, parents’ education level, and economic status of the family would have an impact on incidence of TDI.

## 2. Materials and Methods

The questionnaire surveys among 18-year-old adolescents were carried out in 2020 as part of the program of the Ministry of Health called “Monitoring the health of the oral cavity of the Polish population in 2016–2020”. The study was approved by the Bioethics Committee at the Medical University of Warsaw (KB/134/2017 of 6 June 2017). 

The research was conducted nationwide and covered 15 voivodeships. The sample of the population was selected in a multi-layered grouping. In each of the voivodeships, powiats, communes, towns, villages, and schools in individual towns were randomly selected. Written consent was obtained from school principals to conduct the study and those invited to participate in the study. Only those people who completed the questionnaire and signed consent to conduct the study were included in the study.

Using the data from the Central Statistical Office on the number of 18-year-olds in Poland and in individual provinces, the proportional sizes of the groups for individual provinces were estimated [15]. The sample size was calculated to account for a 3% standard error, 99% confidence interval (CI), and 85% test power. Based on a study of 15-year-old adolescents in Poland, the TDI incidence rate was estimated at 22% [16]. The sample size necessary to meet these requirements was estimated at 1201 people, and the minimum number estimated based on the frequency of permanent tooth injuries was 881 (22%) (95% CI, 18.2–27.4%). The minimum number of questionnaires necessary for the analysis was 1500. Taking into account the possibility of respondents’ refusal and the specific working conditions of schools during the COVID-19 pandemic (the possibility of remote or hybrid learning), it was decided to increase the number of invitations to the study three-fold.

Questionnaires for monitoring studies were prepared in accordance with the criteria of the World Health Organization [17]. They included sections on socio-economic factors, oral health behavior, oral health self-assessment and related quality of life, and questions on permanent tooth injuries prepared on the basis of a questionnaire used in studies of 15-year-olds, such as [16]:

(i) number and location of injured teeth;
(ii) type of injury: tooth luxation (e.g., subluxation, lateral luxation, extrusion, intrusion), avulsion, fracture of the crown;
(iii) accompanying wounds of soft tissues (face skin, lips, gums, tongue);
(iv) applied treatment (splinting of teeth, root canal treatment, reconstruction of a broken crown, tooth extraction, no need for treatment);
(v) complications of trauma (tooth loss, discoloration);
(vi) causes of the injury (violence, accidental injury: sports-related (e.g., cycling, swimming, running), car accident, other activity (e.g., play)) and the place where the injury occurred (school or outside school).

In order to assess the type of traumatic injury, terms understandable to non-professionals were used: increased tooth mobility, displacement of the tooth in any lateral direction or elongated tooth, tooth driven into the gum/bone, and tooth knocked out. Displaced teeth, elongated teeth, and increased mobility of teeth were diagnosed as injuries, such as extrusion, subluxation, and lateral luxation, and we defined them as dislocation, tooth driven into the gum/bone as intrusion, and tooth knocked out as avulsion. 

Two subgroups were formed in the luxation group: the first included intrusion, and the second contained subluxation, lateral luxation, and extrusion. Teeth reported as displaced or elongated and loose teeth were included in the second group.

Due to the full-time or hybrid teaching mode during the pandemic, written information about the nature of the research and paper and electronic questionnaires were prepared in various regions of Poland. The respondents were asked by the teacher to fill in the questionnaires during lesson hours (45 min). About 80% of the questionnaires were completed on paper. Researchers entered the data into an electronic database.

In the statistical analysis, in order to compare the significance of the differences between the values of the studied parameters depending on gender, the test for two proportions (Z test) based on the chi-square distribution was used. The relationship between the analyzed parameters and socio-economic variables was assessed using Spearman’s rank correlation analysis. Based on simple and multiple logistic regression analysis, odds ratios (ORs) and adjusted odds ratios (AORs) were determined together with 95% confidence interval (CI). The significant factors were socio-economic factors (gender, parents’ level of education, self-assessed economic status of the family) and the type of injury. The level of significance was *p* ≤ 0.05.

## 3. Results

A total of 120 schools were invited to participate in the research. The study was approved by principals of 88 schools, where the lessons were conducted in a full-time or hybrid mode. Ultimately, 1741 questionnaires completed by 18-year-olds were eligible for analysis, including 1062 female and 679 male respondents. 

Among the respondents, tooth trauma was reported by 18% of adolescents, with a similar frequency in males and females (Table 1). The most common trauma occurred during other activities (201/315, 64%). Beating as the cause of injury was reported by only 22 respondents (22/315, 7%). One in four respondents reported that the injury was related to playing sports (80/315, 25%). The most common direct cause of injury was a fall (134/315, 43%) or a collision with an object or another person (112/315, 36%). In most cases, the injury happened at school. In 60% (188/315) of cases, the trauma was related to a single tooth. Nearly half (142/315, 45%) of the injuries concerned the anterior teeth of the maxilla, followed by the posterior teeth of the mandible (81/315, 26%). In 56% (176/315) of all reported cases, the injuries were related to a tooth crown fracture (Table 1).

Treatment of traumatic injuries was reported by 74% (233/315) of those with injuries. Of the 139 respondents reporting any type of luxation injury, splinting of teeth was performed in 26 (19%). No need for treatment, root canal treatment, and splinting were correlated with number of injured teeth (r: −0.1667, 0.1709, 0.1819, respectively, *p* < 0.0001). Eighty-one respondents (5% of the entire study population, 4% of females, and 5% of males; *p* = 0.2399) reported tooth loss due to trauma (48 immediately after the injury). Of the remaining 234 respondents, 68 (29%) reported tooth discoloration after trauma. In 26% (81/315) of all dental trauma cases, including 25% of females and 27% of males, a tooth was lost due to injury.

In the next stage, the correlations between type of injury (breaking the crown, subluxation, lateral luxation, extrusion, intrusion, avulsion) and its cause were investigated (Table 2). In the study group, in all cases (beatings, sports activities, car accidents, and other activities), tooth fracture was the most common injury. A significant relationship between type of injury and its cause was identified in the case of beating and sports activity. Crown fracture was also the most common type of injury for all the analyzed direct causes of injury.

Correlations between type of injury and treatment method are presented in Table 3. In the case of intrusion, root canal treatment was most often used, while subluxation, lateral luxation, and extrusion did not require further treatment. Soft tissue injuries (skin of the face, lip, gum, tongue) were accompanied by crown fractures in 43 cases (51%) and least frequently during intrusion (10/84, 12%) and avulsion (11/84, 13%).

Statistically significant relationships between the event that led to the injury and its immediate cause were also demonstrated (Table 4).

Spearman correlation analysis did not confirm a relationship between any of the trauma parameters and living in a rural or urban region. However, it did demonstrate the importance of socio-economic situation and socio-economic factors in the studied group (gender of the respondent, level of parents’ education, and the financial situation of the family) (Table 5). 

Male gender of the respondents was positively associated with the event that led to the injury (sports activity and beating) and no need for treatment, and it was negatively associated with root canal treatment. The financial situation of the family influences application of the treatment method. Logistic regression analysis did not confirm the relationship between injury and male gender. Risk of injury was increased by a low level of parents’ education and poor financial situation of the family. None of the analyzed socio-economic factors increased the chance of injury as a result of a specific event or lack of treatment. A low level of education of the mother increased the chance of losing a tooth and not restoring the tooth crown.

## 4. Discussion

The questionnaire studies illustrate the problem of permanent tooth injuries in Poland and the factors related to occurrence of an injury and its treatment. The first national cross-sectional study of frequency of injuries in adolescents was conducted in Poland in 2018 in a group of 15-year-olds [16]. In this study, TDI were found in 22% of respondents. However, the study was based on both clinical evaluation of the dentition and information collected using questionnaires filled in by adolescents. The current study presents only the self-reported experiences of adolescents regarding the experience of a permanent tooth injury. Due to the COVID-19 pandemic, the study relied solely on information collected through a questionnaire, so no clinical data from patients were collected. Our respondents were asked to report on the diagnosis that was more serious in their opinion. This type of research, especially among young people, requires formulation of questions in such a way that they are understandable to the respondents. This prevented the authors from gathering accurate information on the types of injuries included in professional classifications and used in clinical trials [3,4,11,12,13,18,19]. A research methodology similar to that of the present study has been reported by other researchers [20]. Their questionnaire, similar to the one used in the present study, was designed to report and analyze one episode and type of injury, which made it impossible to record data from respondents who experienced an injury more than once. Therefore, when analyzing the test results, it should be remembered that, in about 10% of permanent teeth, more than one lesion is diagnosed [21].

The strengths of the current study are the assured anonymity, the large size of the study group, and the request to report an injury regardless of whether treatment was started. As is well known, many injuries are not considered to require treatment. On the other hand, the research was limited by the COVID-19 pandemic and the possibility of asking only questions that were understandable to the respondents. This, in turn, made detailed classification of injuries difficult.

It is difficult to interpret the data on the causes of injuries given by different authors due to their overlapping classifications [3]. Direct causes that were identified in our research, such as hitting an object, a collision with an object or a person, or a fall, may be the result of various activities. For example, hitting a ball is a collision with an object, but it is simultaneously related to sport. Likewise, a fall may be the result of, for example, stumbling during sports or being pushed in the event of violence [18,22,23,24]. The present analysis showed that sport is positively correlated with a fall and negatively correlated with object collision. Analysis by Škaričić et al. showed that 34% of injuries caused by collisions with objects and 28% of bicycle/rolling accidents were related to sport [25]. It is extremely important to standardize the methodology of assessing the causes of tooth injuries, that is, to separate the events that lead to them from the immediate causes. A thorough analysis of the cause/site of traumatic tooth injuries was performed by Oldin et al., stating that, in the event of a fall, the most common mechanism of injury is hitting the floor [26].

According to the literature review, the most common location of injuries is the home [27,28]. Our respondents mentioned school as the site of tooth injuries, which is not consistent with the results of other studies. However, this does not change the fact that permanent tooth injuries are often related to sports activities and often occur at school. This emphasizes the need to educate trainers and teachers as well as the students themselves in terms of both injury prevention and first aid. It is also worth recalling that, during adolescence, conflicts with peers and sensitivity to stressful situations intensify. Teenagers are emotionally unstable, which can lead to aggressive behavior [29]. Therefore, comprehensive measures are also necessary to help young people develop mature psychosocial skills, including coping with frustration and stress, which will reduce the risk of aggressive behavior.

It should be noted that not all people who experience Ie tooth trauma consider it necessary to see a dentist, and not all injuries require treatment. In this research, 26% of respondents reported no need for treatment in their self-assessment. In a Brazilian study, only 26% of adolescents visited a dentist after an injury, which is consistent with our results [24]. Moreover, it is worth noting that, in the study group, socio-economic factors were associated with a subjective assessment of a lack of need for treatment and loss of the tooth/teeth. This means that youth of low socio-economic status less often visit a dentist.

One in five respondents developed tooth discoloration after injury, and, for one in four, the injury was the cause of tooth loss. The percentage of teeth discolored by trauma reported by Francisco et al. was significantly lower (5%) [30]. Tooth injuries are at risk of developing various complications, such as pulp necrosis, obliteration of the pulp cavity, and root and bone resorption, which may result in tooth discoloration or loss [31]. Their occurrence depends on many factors, including those related to the injury itself, its treatment, or the stage of tooth development. Retrospective studies of 531 medical records showed that male gender and tooth avulsion increase the chance of complications 2.79- and 7.53-fold, respectively [32]. In the cited studies, post-traumatic complications (most often necrosis) affected as many as 50% of teeth. In the groups studied, no influence of gender on the occurrence of complications was noticed. Although significant correlations were found between male gender and no need for treatment, logistic regression did not confirm a lower chance of treatment in males, even after introduction of type of trauma and socio-economic factors as confounding factors. It also did not confirm the importance of gender in the type of treatment of traumatic injuries. In our group, injuries most often caused damage to the anterior teeth of the maxilla, and, in most cases, only one tooth was affected; these results agree with other studies [7,10,11,18,19].

Only a few researchers recorded soft tissue injuries. The literature reports a wide range of incidences of oral soft tissue injuries, from 3.5% to nearly half of all dental traumas [25,33]. Soft tissue damage is less likely to occur when fewer teeth were damaged, which is in line with our results. Likewise, it was also found that lesions of the lips were most frequent, followed by the gums and tongue. In our results, soft tissue injuries were positively correlated with tooth avulsion and negatively correlated with crown fracture. Similarly, in a study by Škaričić et al., soft tissue injuries were more often associated with injuries to periodontal tissue than injuries to hard dental tissue and pulp [25].

Many researchers have analyzed the relationship between occurrence of tooth trauma and gender, showing a higher incidence of trauma in males compared to females [3,4,5]. In a study by Huang et al., tooth trauma occurred in 12% of males and only 8% of females [27]. The relationship with gender was justified primarily by greater sports activity and competition among boys and lower maturity compared to girls [3]. However, in the group we studied, the difference in the incidence of injuries among males and females was not statistically significant. Similarly, other authors also did not observe any relationship with gender [20,26]. Traebert et al. argue that modern females living in Western societies may be exposed to the same risk factors as males [18].

Socio-economic factors may have a multi-level impact on causality of tooth injuries, availability of dental care, and the treatment procedure [11,12,24]. However, this relationship is not noted by all authors [14]. This variation can be explained by the lack of methodological uniformity. In studies on this issue, various indicators of socio-economic status have been used, including type of school (public or private), level of education of the father or mother, monthly income, and the social sensitivity index [10,24]. The differences in results on the incidence of dental injuries obtained in different regions of the world also depend on the influence of other factors, including geographic, economic, and cultural. Our research presents self-reported evidence from adolescents, both on experiencing permanent tooth trauma and socio-economic factors. It is worth emphasizing that not all of our respondents were able to answer the questions about the level of their parents’ education. An additional limitation was the risk of “subjective assessment”, as well as the desire to better present one’s family. In our research, we noted the importance of socio-economic factors reported by the respondents. Their impact was confirmed by both Spearman’s analysis and logistic regression. Parents’ low level of education and poor financial situation of the family increased the probability of tooth trauma by approximately 1.5-fold. A higher prevalence of TDI has also been reported among British teenagers of low socio-economic status [32].

## 5. Conclusions

This study shows comparable incidence of permanent tooth injuries in the reports of Polish youth and in epidemiological studies. Playing sports is important in the etiology of injuries. Many of the injuries occurred in school, demonstrating the need for school education in injury prevention and first aid. The research confirms the hypothesis that parents’ low level of education and poor financial situation significantly increase level of tooth trauma. However, there were no differences in incidence of injuries between men and women. The research demonstrates the need for universal education on treatment of dental injuries.

## Figures and Tables

**Table 1:** Occurrence, type, causes, and place of trauma to permanent tooth/teeth, taking into account gender.

Parameter		Total *N* = 1741	Females *n* = 1062	Males *n* = 679	*p*
People with an injury		315/1741 (18%)	182/1062 (17%)	133/679 (20%)	0.1585
Cause of the injury	Beating	22/315 (7%)	8/182 (4%)	14/133 (11%)	0.1083
	Sports activities	80/315 (25%)	40/182 (22%)	40/133 (30%)	0.2646
	Car accident	12/315 (4%)	7/182 (4%)	5/133 (4%)	0.9992
	Other activity	201/315 (64%)	127/182 (70%)	74/133 (56%)	0.0359
Immediate cause	Hit with an object	69/315 (22%)	41/182 (23%)	28/133 (21%)	0.9523
	Fall	134/315 (43%)	81/182 (45%)	53/133 (40%)	0.7112
	Collision with an object or a person	112/315 (36%)	60/182 (33%)	52/133 (40%)	0.5325
Place where the injury occurred	School	271/315 (86%)	160/182 (88%)	111/133 (83%)	0.5340
	Outside of school	44/315 (14%)	22/182 (12%)	22/133 (17%)	0.5340
Number of injured teeth	1	188/315 (60%)	101/182 (56%)	87/133 (65%)	0.2078
	2	78/315 (25%)	46/182 (25%)	32/133 (24%)	0.9533
	>2	49/315 (16%)	35/182 (19%)	14/133 (10%)	0.1098
Location of the tooth with the trauma	Posterior upper	66/315 (21%)	40/182 (22%)	26/133 (20%)	0.8721
	Anterior upper	142/315 (45%)	74/182 (41%)	68/133 (51%)	0.1825
	Posterior lower	81/315 (26%)	53/182 (29%)	28/133 (21%)	0.2766
	Anterior lower	26/315 (8%)	15/182 (8%)	11/133 (8%)	0.9987
Type of injury	Subluxation, lateral luxation, Extrusion	80/315 (25%)	50/182 (28%)	30/133 (23%)	0.6126
	Intrusion	31/315 (10%)	21/182 (12%)	10/133 (8%)	0.4967
	Avulsion	28/315 (9%)	12/182 (7%)	16/133 (12%)	0.2461
	Breaking the crown	176/315 (56%)	99/182 (55%)	77/133 (58%)	0.8263
Injury of soft tissues	Skin of the face	10/135 (3%)	7/182 (4%)	3/133 (2%)	0.7289
	Lip	29/315 (9%)	12/182 (7%)	17/133 (13%)	0.1442
	Gum	41/315 (13%)	24/182 (13%)	17/133 (13%)	0.9945
	Tongue	4/315 (1%)	2/182 (1%)	2/133 (2%)	0.9510
Post-traumatic complications	Discoloration	68/315 (22%)	43/182 (24%)	25/133 (19%)	0.3733
	Tooth loss	81/315 (26%)	45/182 (25%)	36/133 (27%)	0.7344

**Table 2:** Correlations between type of injury and cause of injury (Spearman’s r-correlation coefficient).

Cause of the Injury	Type of Injury											
	Breaking the Crown			Subluxation, Lateral Luxation, Extrusion			Intrusion			Avulsion		
Incident												
	*N*	r	*p*	*N*	r	*p*	*N*	r	*p*	*N*	r	*p*
Beating	9/22 (41%)	−0.0826	0.0211	7/22 (32%)	0.0404	0.1281	3/22 (14%)	0.0349	0.1099	3/22 (14%)	0.0457	0.0661
Sports activities	42/80 (53%)	−0.0557	0.0487	16/80 (20%)	−0.0696	0.0445	9/80 (11%)	0.0353	0.0991	13/80 (17%)	0.1693	<0.0001
Car accident	8/12 (67%)	0.0404	0.1928	3/12 (25%)	0.0004	0.9191	1/12 (8%)	−0.0086	0.6152	0/12 -	−0.0631	0.0102
Other activity	117/201 (58%)	0.0362	0.2718	54/201 (27%)	0.0667	0.0322	18/201 (9%)	−0.0302	0.1191	12/201 (6%)	−0.1356	<0.0001
The immediate cause of injury												
Hit with an object	46/69 (67%)	0.1151	<0.0001	16/69 (23%)	−0.0269	0.2091	4/69 (6%)	−0.0719	0.0211	3/69 (4%)	−0.0845	0.0291
Collision with an object or a person	63/112 (56%)	0.0056	0.1921	24/112 (21%)	−0.0677	0.0401	14/112 (13%)	0.0663	0.0488	11/112 (10%)	0.0243	0.2009
Fall	67/134 (50%)	−0.1018	<0.0001	40/134 (30%)	0.0880	0.0031	13/134 (10%)	−0.0040	0.7781	14/134 (10%)	0.0471	0.3918

**Table 3:** Correlations between type of injury and treatment method used (Spearman’s r-correlation coefficient).

Type of Injury												
Treatment	Breaking the Crown			Subluxation, Lateral Luxation, Extrusion			Intrusion			Avulsion		
Root canal treatment	25/176 (14%)	−0.1866	<0.0001	29/80 (36%)	0.2193	<0.0001	12/31 (39%)	0.1442	<0.0001	0/28 -	−0.1608	<0.0001
Tooth extraction	26/176 (15%)	−0.0146	0.4099	10/80 (13%)	−0.0445	0.0881	4/31 (13%)	−0.0215	0.1085	8/28 (29%)	0.1159	<0.0001
No need for treatment	47/176 (27%)	0.0173	0.4112	25/80 (31%)	0.0694	0.0201	6/31 (19%)	−0.0503	0.4551	4/28 (14%)	−0.0836	0.6511
Injury of soft tissues	43/84 (51%)	−0.0569	0.0409	20/84 (24%)	−0.0220	0.2011	10/84 (12%)	0.0418	0.0881	11/84 (13%)	0.0891	0.0321

**Table 4:** Correlations between cause of injury and event (Spearman’s r-correlation coefficient).

Cause of the Injury	An Incident Leading to an Injury											
	Beating			Playing Sport			Car Accident			Other Activity (e.g., Play)		
	*N*	r	*p*	*N*	r	*p*	*N*	r	*p*	*N*	r	*p*
Hit with an object	6/22 (27%)	0.0356	0.1101	10/80 (13%)	−0.1343	<0.0001	3/12 (25%)	0.0176	0.3818	50/201 (25%)	0.1214	<0.0001
Collision with an object or a person	8/22 (36%)	0.0046	0.4415	43/80 (54%)	−0.0223	0.2918	5/12 (42%)	−0.0093	0.7691	78/201 (39%)	0.0254	0.1029
Fall	8/22 (36%)	−0.0342	0.3321	27/80 (34%)	0.1330	<0.0001	4/12 (33%)	−0.0056	0.8889	73/201 (36%)	−0.1253	<0.0001

**Table 5:** Results of simple and multiple logistic regression analyses.

Parameter	OR/AOR		Cause of the Injury			Treatment			Tooth Loss Due to Trauma
			Beating	Playing Sport	Other Activity (e.g., Play)	Root Canal Treatment	Restoration of the Crown	No Need for Treatment	
Males	r (*p*)	0.0977 (0.0010) *	0.1188 (<0.0001) *	0.1171 (<0.0001) *	0.1143 (<0.0001) *	−0.1874 (<0.0001) *	0.0385 (0.1091)	0.1081 (<0.0001) *	0.0265 (0.2301)
	OR (Cl) *p*	1.01 (0.79–1.29) 0.9461	0.94 (0.38–2.24) 0.8815	0.89 (0.53–1.49) 0.6592	1.16 (0.71–1.91) 0.5568	1.08 (0.63–1.86) 0.7835	1.02 (0.63–.65) 0.9399	0.97 (0.58–1.60) 0.8922	1.22 (0.73–2.02) 0.4475
	AOR 1 (Cl) *p*	1.01 (0.79–1.29) 0.9176	0.98 (0.40–2.38) 0.9617	0.89 (0.53–1.50) 0.6717	1.16 (0.70–1.91) 0.5712	1.08 (0.62–1.86) 0.7863	1.02 (0.62–1.66) 0.9523	0.97 (0.58–1.60) 0.8988	1.22 (0.74–2.04) 0.4353
	AOR 2 (Cl) *p*	1.03 (0.81–1.32) 0.8099	1.00 (0.41–2.41) 0.9968	0.89 (0.53–1.49) 0.6494	1.15 (0.70–1.90) 0.5709	1.12 (0.65–1.94) 0.6875	0.95 (0.58–1.56) 0.8465	0.99 (0.59–1.65) 0.9725	1.28 (0.77–2.14) 0.3493
	AOR 3 (Cl) *p*	1.03 (0.81–1.32) 0.8035	1.00 (0.40–2.44) 0.9972	0.88 (0.52–1.48) 0.6376	1.16 (0.71–1.93) 0.5543	1.12 (0.65–1.95) 0.6799	0.96 (0.58–1.58) 0.8819	0.99 (0.59–1.65) 0.9687	1.27 (0.76–2.13) 0.3539
Father’s level of education	r (*p*)	−0.1078 (0.0087) *	−0.0090 (0.1920)	−0.0087 (0.3991)	0.0280 (0.6556)	−0.0619 (0.3341)	0.0769 (0.0018) *	0.0269 (0.1101)	−0.0426 (0.6610)
Father’s low level of education	OR (Cl) *p*	1.39 (1.08–1.77) 0.0093	1.71 (0.72–4.27) 0.2304	0.96 (0.57–1.61) 0.8772	0.94 (0.57–1.55) 0.8152	1.10 (0.64–1.89) 0.7392	0.63 (0.38–1.03) 0.0679	0.98 (0.59–1.63) 0.9453	1.41 (0.85–2.36) 0.1800
	AOR 1 (Cl) *p*	1.37 (1.07–1.76) 0.0113	1.52 (0.62–3.86) 0.3576	0.92 (0.54–1.55) 0.7493	1.00 (0.60–1.65) 0.9907	1.11 (0.64–1.91) 0.7154	0.67 (0.41–1.11) 0.1233	0.97 (0.58–1.61) 0.8991	1.38 (0.83–2.30) 0.2168
	AOR 2 (Cl) *p*	1.34 (1.05–1.72) 0.0198	1.59 (0.66–4.00) 0.3049	0.96 (0.57–1.63) 0.8925	0.95 (0.58–1.57) 0.8456	1.05 (0.60–1.82) 0.8710	0.69 (0.41–1.13) 0.1450	0.95 (0.56–1.58) 0.8298	1.34 (0.80–2.25) 0.2602
	AOR 3 (Cl) *p*	1.34 (1.04–1.72) 0.0210 *	1.49 (0.61–3.80) 0.3834	0.93 (0.55–1.58) 0.8018	0.99 (0.60–1.64) 0.9623	1.06 (0.61–1.84) 0.8415	0.72 (0.43–1.19) 0.1981	0.94 (0.56–1.57) 0.8119	1.33 (0.79–2.23) 0.2805
Mother’s level of education	r (*p*)	−0.0905 (0.0012) *	−0.0762 (0.0455) *	0.0153 (0.4821)	0.0071 (0.8878)	−0.0523 (0.0409) *	0.0848 (0.1021)	0.0955 (0.0038) *	−0.1519 (<0.0001) *
Mother’s low level of education	OR (Cl) *p*	1.62 (1.25–2.11) 0.0003 *	2.39 (1.00–5.84) 0.0509	0.87 (0.50–1.50) 0.6253	0.98 (0.59–1.67) 0.9514	1.08 (0.61–1.89) 0.7821	0.51 (0.29–0.87) 0.0150 *	0.82 (0.48–1.40) 0.4782	2.13 (1.27–3.58) 0.0041 *
	AOR 1 (Cl) *p*	1.60 (1.23–2.08) 0.0005 *	1.95 (0.79–4.89) 0.1463	0.82 (0.46–1.42) 0.4758	1.07 (0.63–1.83) 0.8102	1.10 (0.61–1.94) 0.7463	0.56 (0.32–0.96) 0.0375 *	0.80 (0.46–1.37) 0.4214	2.07 (1.23–3.51) 0.0063 *
	AOR 2 (Cl) *p*	1.57 (1.20–2.05) 0.0008 *	2.21 (0.91–5.49) 0.0789	0.87 (0.49–1.52) 0.6361	1.00 (0.59–1.70) 0.9853	1.02 (0.57–1.81) 0.9334	0.56 (0.32–0.96) 0.0385 *	0.78 (0.45–1.34) 0.3781	2.04 (1.20–3.45) 0.0080 *
	AOR 3 (Cl) *p*	1.56 (1.19–2.04) 0.0012 *	1.92 (0.77–4.85) 0.1622	0.83 (0.47–1.45) 0.5173	1.06 (0.62–1.83) 0.8369	1.05 (0.58–1.85) 0.8800	0.59 (0.33–1.02) 0.0643	0.77 (0.44–1.33) 0.3533	2.01 (1.18–3.41) 0.0099 *
Economic situation of the family	r (*p*)	−0.1694 (0.0007) *	−0.0667 (0.0444) *	0.0310 (0.1128)	0.0062 (0.7098)	−0.0829 (0.0149) *	0.1450 (<0.0001) *	−0.053 (0.0455) *	−0.0787 (0.0109) *
Bad or very bad economic situation of the family	OR (Cl) *p*	1.42 (1.04–1.93) 0.0265 *	1.93 (0.71–4.81) 0.1708	0.94 (0.48–1.78) 0.8520	0.99 (0.54–1.88) 0.9740	1.34 (0.69–2.52) 0.3738	0.54 (0.27–1.03) 0.0732	1.26 (0.68–2.29) 0.4559	1.42 (0.76–2.56) 0.2577
	AOR 1 (Cl) *p*	1.38 (1.00–1.89) 0.0472 *	1.35 (0.47–3.56) 0.5531	0.83 (0.41–1.61) 0.5974	1.15 (0.61–2.24) 0.6653	1.39 (0.70–2.67) 0.3272	0.65 (0.32–1.25) 0.2126	1.22 (0.64–2.26) 0.5255	1.33 (0.70–2.44) 0.3699
	AOR 2 (Cl) *p*	1.43 (0.76–2.66) 0.2578	1.57 (0.16–2.39) 0.6782	0.91 (0.24–3.27) 0.8872	1.28 (0.37–4.58) 0.6960	0.89 (0.22–3.44) 0.8725	1.17 (0.34–3.90) 0.8037	0.95 (0.26–3.31) 0.9365	0.79 (0.21–2.82) 0.7251
	AOR 3 (Cl) *p*	1.40 (0.75–2.61) 0.2895	1.51 (0.14–2.82) 0.7168	0.90 (0.24–3.28) 0.8783	1.30 (0.37–4.72) 0.6852	0.90 (0.22–3.47) 0.8831	1.23 (0.35–4.13) 0.7436	0.94 (0.26–3.30) 0.9291	0.78 (0.21–2.79) 0.7103

R—Spearman’s correlation coefficient, OR—odds ratio, AOR 1—confounders: type of injury (subluxation, lateral luxation, extrusion, intrusion, and avulsion), AOR 2—confounders: socioeconomic factors (gender, parents’ level of education, self-assessed economic status of the family), AOR 3—confounders: type of injury and socioeconomic factors; * statistical significance *p* < 0.05.

## Data Availability

The data presented in this study are available on request from the corresponding author. The data are not publicly available due to including personal data of respondents.
